# Dampening of population cycles in voles affects small mammal community structure, decreases diversity, and increases prevalence of a zoonotic disease

**DOI:** 10.1002/ece3.3074

**Published:** 2017-06-09

**Authors:** Frauke Ecke, David G. Angeler, Magnus Magnusson, Hussein Khalil, Birger Hörnfeldt

**Affiliations:** ^1^ Department of Wildlife, Fish, and Environmental Studies Swedish University of Agricultural Sciences Umeå Sweden; ^2^ Department of Aquatic Sciences and Assessment Swedish University of Agricultural Sciences Uppsala Sweden

**Keywords:** bank vole, common shrew, dilution effect, field vole, gray‐sided vole, hantavirus

## Abstract

Long‐term decline and depression of density in cyclic small rodents is a recent widespread phenomenon. These observed changes at the population level might have cascading effects at the ecosystem level. Here, we assessed relationships between changing boreal landscapes and biodiversity changes of small mammal communities. We also inferred potential effects of observed community changes for increased transmission risk of Puumala virus (PUUV) spread, causing the zoonotic disease nephropatica epidemica in humans. Analyses were based on long‐term (1971–2013) monitoring data of shrews and voles representing 58 time series in northern Sweden. We calculated richness, diversity, and evenness at alpha, beta, and gamma level, partitioned beta diversity into turnover (species replacement) and nestedness (species addition/removal), used similarity percentages (SIMPER) analysis to assess community structure, and calculated the cumulated number of PUUV‐infected bank voles and average PUUV prevalence (percentage of infected bank voles) per vole cycle. Alpha, beta, and gamma richness and diversity of voles, but not shrews, showed long‐term trends that varied spatially. The observed patterns were associated with an increase in community contribution of bank vole (*Myodes glareolus*), a decrease of gray‐sided vole (*M. rufocanus*) and field vole (*Microtus agrestis*) and a hump‐shaped variation in contribution of common shrew (*Sorex araneus*). Long‐term biodiversity changes were largely related to changes in forest landscape structure. Number of PUUV‐infected bank voles in spring was negatively related to beta and gamma diversity, and positively related to turnover of shrews (replaced by voles) and to community contribution of bank voles. The latter was also positively related to average PUUV prevalence in spring. We showed that long‐term changes in the boreal landscape contributed to explain the decrease in biodiversity and the change in structure of small mammal communities. In addition, our results suggest decrease in small mammal diversity to have knock‐on effects on dynamics of infectious diseases among small mammals with potential implications for disease transmission to humans.

## 1 INTRODUCTION

The global loss of biodiversity has become a major concern as it negatively affects ecosystem functioning and potentially alters the emergence and transmission of infectious diseases (Balvanera et al., 2006; Hooper et al., 2012; Keesing et al., 2010; Loreau et al., 2001). The concept of the dilution effect predicts that a high proportion of noncompetent reservoirs/hosts (i.e., so‐called dead ends) occurring in diverse animal communities reduces disease risk by, for example, reducing encounter rates of competent hosts, thereby reducing transmission risk among these (Ostfeld & Keesing, 2000; Schmidt & Ostfeld, 2001). High biodiversity is therefore expected to contribute to ecosystem and human health (Keesing et al., 2010).

**Figure 1:**
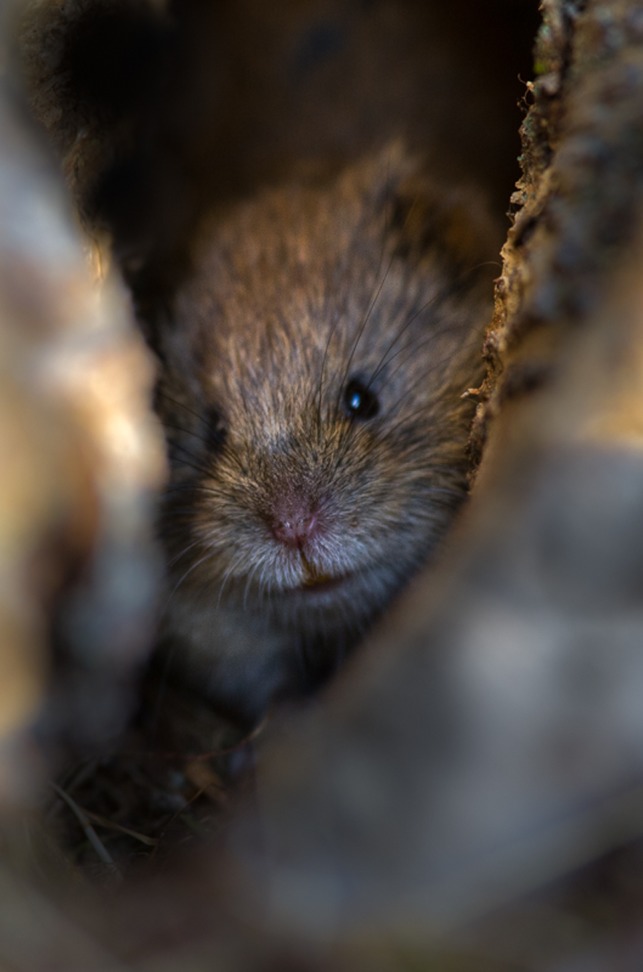
Vole in a stone hole (Photo credit: Rolf Segerstedt)

Small mammals (Figure 1) are key species for the functioning of many ecosystems. They serve as staple food for many mammalian and avian predators (Englund, 1970; Erlinge, 1987; Hörnfeldt, 1991; Lindström, 1982), consume plants and invertebrates, and disperse seeds and fungi (Ericson, 1977; Gebczynska, 1983; Hansson, 1998; Terwilliger & Pastor, 1999). In addition, small mammals are reservoirs, hosts, and vectors of many socioeconomically important zoonotic (animal spread) diseases (Han, Schmidt, Bowden, & Drake, 2016; Matuschka, Fischer, Musgrave, Richter, & Spielman, 1991; Nichol et al., 1993; Sjöstedt, 2007; Vapalahti et al., 2003). Pathogens hosted by small mammals and transmitted to humans include the Puumala virus (PUUV) hosted by the bank vole (*Myodes glareolus* Schreber) (Brummer‐Korvenkontio et al., 1980) and causing *nephropatia epidemica* (NE), a hemorrhagic fever in humans, sometimes leading to kidney failure, and in rare cases to death (Vapalahti et al., 2003). The annual incidence of NE in humans can be explained with high degree (84% of variability) by density of *M. glareolus* in autumn and the number of rainy days in winter (Khalil, Olsson, et al., 2014). In contrast, the number of PUUV‐infected bank voles and PUUV prevalence (the proportion of infected bank voles) has been shown to be affected by habitat type (Magnusson, Ecke, et al., 2015; Voutilainen et al., 2012).

**Figure 2:**
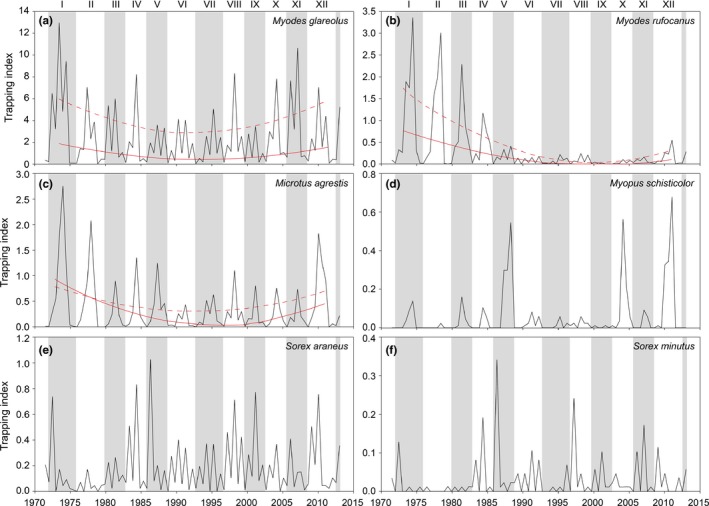
Density of the six most common small mammal species in the study area near Umeå (a‐f), as revealed by trapping indices in spring and autumn in 1971–2013 from autumn 1971, and representing 12 complete cycles (I–XII) of which every second cycle is indicated by shading. Note the different scales of the trapping index for the respective species. Fitted lines show significant changes in the trapping index in spring (continuous lines) and autumn (dashed lines) for the three vole species. See S1 for the statistical analyses

During the last decades, long‐term decline and depression of density in cyclic voles has become a widespread phenomenon (Cornulier et al., 2013; Hörnfeldt, 2004; Ims, Henden, & Killengreen, 2008). The causes of these declines are largely unknown, but probably involve multiple elements including climatic factors (Cornulier et al., 2013; Kausrud et al., 2008; Korpela et al., 2013, 2014) and, at least in Sweden, landscape changes (Magnusson, Hörnfeldt, & Ecke, 2015). The small mammal‐related changes not only comprise altered population dynamics (Cornulier et al., 2013; Hörnfeldt, 2004) but in one case also local and regional disappearance of species (Hörnfeldt, Christensen, Sandström, & Ecke, 2006). In northern Sweden, especially the forest dwelling gray‐sided vole (*Myodes rufocanus* Sundevall) and the open habitat specialist field vole (*Microtus agrestis* Linnaeus), but also the largely forest dwelling bank vole showed ongoing patterns of decline since the mid‐1970s (Figure 2; see also Ecke, Magnusson, & Hörnfeldt, 2013; Hörnfeldt, 2004; Magnusson, Hörnfeldt, & Ecke, 2015).

Despite observed changes in the small mammal population dynamics potentially affecting structural and functional patterns at the ecosystem level (Hörnfeldt, Hipkiss, & Eklund, 2005; Ims et al., 2008; Olofsson, Tommervik, & Callaghan, 2012), analyses of small mammal community structure and biodiversity are lacking. Considering the importance of small mammals for ecosystem functioning in general, and for trophic interactions and disease transmission in particular, any potential long‐term changes in the diversity of small mammals might have profound effects for ecosystem and human health. In fact, according to the concept of the dilution effect, a decrease in small mammal diversity should increase disease risk (Ostfeld & Keesing, 2000; Schmidt & Ostfeld, 2001). In a most recent study, temporal changes in PUUV infection risk in bank voles were related to changes in the density index of shrews and field voles (Khalil, Ecke, Evander, Magnusson, & Hörnfeldt, 2016).

The aim of this study is fivefold. First, using long‐term (1971–2013) monitoring data representing 58 time series, we test whether the observed declines in the population density of voles also imply a decline, or other changes of biodiversity. Because impacts of environmental change can be scale specific (Angeler & Drakare, 2013), we study biodiversity patterns at the alpha, beta, and gamma level, using multiplicative partitioning (Whittaker, 1960, 1972), which achieves mathematical independence between alpha and beta diversity (Baselga, 2010). Alpha biodiversity refers to the local (within habitats) scale, whereas beta biodiversity relates to differences in community composition between habitats and gamma biodiversity to the regional scale. We assess these changes for taxon richness and diversity (exponential Shannon entropy) that cover distinct ecological phenomena, with richness focusing only on species presence–absence patterns and diversity covering both species abundances and incidences (Tuomisto, 2012). We also study evenness patterns of the small mammal communities to assess how species dominance patterns affect diversity. Second, by partitioning beta diversity into turnover and nestedness (Baselga & Orme, 2012), we infer the relative role of contrasting processes influencing long‐term beta diversity, that is, of species replacement (turnover) versus species addition/removal (nestedness). Third, considering the importance of landscape structure for the population dynamics of small mammals (see above and Ecke et al., 2010; Magnusson, Hörnfeldt, & Ecke, 2015), we test whether temporal changes in alpha, beta, and gamma richness and diversity as well as in partitioned beta diversity components can be explained by changes in landscape structure. Fourth, we discuss the implications of our results for ecosystem functioning of the boreal forest system in terms of PUUV dynamics and transmission risk. To achieve this, we analyze if altered disease dynamics (as shown by the cumulated number of PUUV‐infected bank voles and average PUUV‐prevalence per cycle) are related to not only changes in population dynamics of small mammals (as shown in Khalil et al., 2016) but also to changes in their diversity. Considering the increasing evidence for the importance of diversity for pathogen prevalence in, for example, hantavirus systems (Clay, Lehmer, St Jeor, & Dearing, 2009a,b; Dizney & Dearing, 2016; Dizney & Ruedas, 2009; Suzán et al., 2009), we expect that any long‐term changes in small mammal diversity would also be reflected in altered pathogen dynamics. Finally, we identify important fields of future research linking small mammal biodiversity with disease ecology (sensu Johnson, Ostfeld, & Keesing, 2015). Our results strongly suggest decrease in small mammal diversity to have knock‐on effects on dynamics of infectious diseases among small mammals.

## 2 MATERIAL AND METHODS

### 2.1 Small mammal monitoring

The 100 × 100 km study area is located in the middle boreal vegetation zone (Ahti, Hämet‐Ahti & Jalas, 1968) near Umeå, northern Sweden (≈ 63°50′N, 19°50′E) (see, for example, Ecke, Magnussson, & Hörnfeldt, 2013; Hörnfeldt, 1978; Magnusson, Ecke, et al., 2015). In each of 16 5 × 5 km subareas, four 1‐ha trapping plots are placed unless a trapping plot hit water or other untrappable habitat (*n* = 6), resulting in a total of 58 permanent and systematically placed trapping plots. The trapping plots contain 10 trap stations with five traps each, centered and spaced 10 m apart along the diagonal of the 1‐ha square. Small mammals have been snap‐trapped twice per year (spring and autumn) since autumn 1971, each trapping session comprising three trap‐nights (see Hörnfeldt, 1978, 1994 for details of the trapping design and trapping methods). The snap‐trapping method does not affect the trapping success and population dynamics of the small mammals (Christensen & Hörnfeldt, 2003).

All woodmice (*Apodemus* spp.) specimens trapped were treated as yellow‐necked mouse (*Apodemus flavicollis* Melchior) as the current distribution range of both Swedish wood mice species suggests that only yellow‐necked mouse occurred in the study area (Bjärvall & Ullström, 2000). The shrews (*Sorex* spp.) and Eurasian water shrew (*Neomys fodiens* Pennant) occurring in the study area are all insectivorous. The wood lemming (*Myopus schisticolor* Liljeborg), *M. glareolus*,* M. rufocanus,* and *M. agrestis* belong to the subfamily of Arvicolinae rodents and were referred to as voles.

### 2.2 Landscape structure

The study area is dominated by different but mainly coniferous forest types (>70%) with some occurrence of wetlands (9%) (Ecke et al., 2010; Hörnfeldt et al., 2006). Since the 1950s, boreal forests have been subject to intense, mainly forestry induced landscape changes (Esseen, Ehnström, Ericson, & Sjöberg, 1992, 1997). In the western 50 × 100 km part of the study area, this has resulted in a decrease in the mean patch area of forest >50 years old from approx. 90 ha in the 1950s to 10 ha in 2005, whereas the area decreased from 30 to ca. 5 ha in the eastern 50 × 100 km part of the study area (Ecke, Magnussson, & Hörnfeldt, 2013). Today, the study area is dominated by single‐layered and even‐aged coniferous forest (Ecke, Magnussson, & Hörnfeldt, 2013).

### 2.3 Data analyses

We used trapping data from 1971 to 2013. The trapping index (number of trapped specimens per 100 trap‐nights) of each species per trapping plot and trapping session, and here referred to as density, was aggregated per vole cycle using the same classification method as in Magnusson, Hörnfeldt, & Ecke, 2015. A vole cycle generally comprises 3–4 years and is characterized by an increase, peak, decrease, and low phase in vole density (Hörnfeldt, 1994). The transition between successive cycles is characterized by a major shift in rate of change in numbers in summer, from low to high(er) values (Figure 3 in Hörnfeldt, 1994; Figure 8 in Hörnfeldt, 2004). A cycle is defined as the period starting and ending, respectively, in spring of years with subsequent cycles’ initial summer increase (or increase phase) (Hörnfeldt, 1994). We delimited cycles based on the bank vole, gray‐sided vole and field vole, considering the overall increase phase per cycle to start in spring of the year when at least two of these species were in the increase phase. In total, there were 12 complete vole cycles in 1972–2012. To avoid confusion when cumulating densities per cycle, and not “duplicating” spring densities at transition of cycles, cycle I encompasses trapping data 1972–1975, cycle II 1976–1979, cycle III 1980–1982, cycle IV 1983‐1985, cycle V 1986–1988, cycle VI 1989–1992, cycle VII 1993–1996, cycle VIII 1997–1999, cycle IX 2000–2002, cycle X 2003–2005, cycle XI 2006–2008, and cycle XII 2009–2012 (see also Figure 2). Data on PUUV‐antibody positive bank voles in spring and autumn were available for the vole cycles III‐IV (Niklasson, Hörnfeldt, Lundkvist, Björsten, & Leduc, 1995) and X‐XII and as Khalil et al. (2016) we excluded bank voles with maternal antibodies in any cycle from the analyses. We calculated the cumulated number of infected bank voles and average PUUV prevalence per cycle, for spring data, by first aggregating data per cycle from spring of the increase phase/year up to and throughout the year before the next cycle's increase phase/year. The number of PUUV‐infected bank voles is related to human disease risk while PUUV‐prevalence reflects transmission ecology in the vole population. Both indices were related to diversity metrics with correlation analysis (Kendall's tau coefficient).

**Figure 3:**
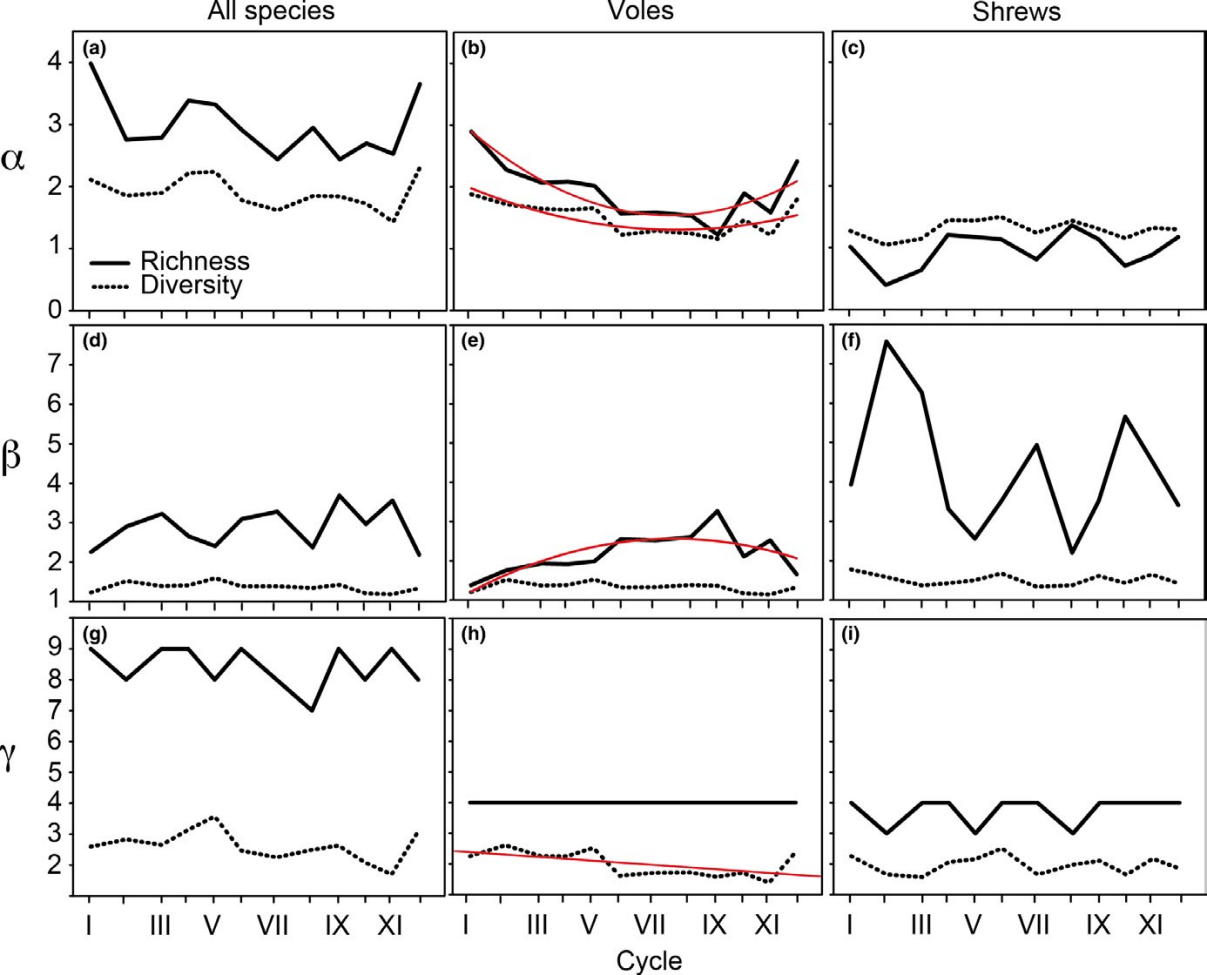
Temporal changes of alpha (a‐c), beta (d‐f), and gamma (g‐i) richness and diversity of all species combined (a, d, g) and separated for voles (b, e, h) and shrews (c, f, i) in the 12 cycles (roman numerals) 1972–2012; cycle I: 1972–1975, II: 1976–1979, III: 1980–1982, IV: 1983–1985, V: 1986–1988, VI: 1989–1992, VII: 1993–1996, VIII: 1997–1999, IX: 2000–2002, X: 2003–2005, XI: 2006–2008, and XII: 2009–2012 (cf. Figure 2). Fitted lines indicate significant temporal changes, tested by polynomial regression (b and e) and linear regression analysis (h) (see Table S2 for the results from the statistical analyses). Distance between tickmarks on the *x*‐axis reflects relative length of cycle (3 or 4 years; cf. Figure 2)

We analyzed the small mammal community structure using metrics that have recently been identified as suitable indices of different biodiversity components. In a first step, we calculated species richness (richness), diversity (exponential Shannon entropy), and evenness following the same approach as Angeler and Drakare (2013). The latter was calculated as the ratio between diversity and richness (Tuomisto, 2012). We calculated these three metrics at the alpha, beta, and gamma level with gamma diversity being the product of alpha and beta diversity (Baselga, 2010). Analyzing all three levels allowed us to infer whether long‐term patterns of biodiversity change of small mammals differ between the local (individual trapping plots) and regional (all 58 trapping plots combined) scale. We also partitioned beta diversity into its turnover (species replacement) and nestedness (species addition/removal) components using the method suggested by Baselga and Orme (2012) where beta diversity is calculated as Sørensen dissimilarity. The dominance of either turnover or nestedness has important implications for nature conservation (Wright & Reeves, 1992). Analysis of dominance allows assessing whether species replacements or extinction patterns require the prioritization of selected habitats or the entire region for management (Angeler, 2013).

To account for abundance of species versus number of species, we tested for differences between richness and diversity using Wilcoxon matched pairs test (Zar, 1996). We used similarity percentage (SIMPER) to a) analyze changes in the similarity of abundances of the small mammals between consecutive cycles, that is, to compare one cycle with the following one, and to b) assess which species contributed most to observed differences between cycles (Clarke, 1993).

Temporal and landscape‐induced changes in richness, diversity, and evenness at the alpha, beta, and gamma level and in beta diversity components were quantified with nonparametric correlation analyses (Spearman's rank correlation) or with first‐order polynomial regressions in case of nonlinearity. We kept the order (*k*) of the polynomial regression low (first order) to avoid over‐fitting. As a measure of landscape change, we used the cumulated area of cutover forests since 1970, that is, forests <50 years old, within 2.5 × 2.5 km large landscapes centered on the individual 58 trapping plots (Ecke, Magnussson, & Hörnfeldt, 2013). The increase in area of this forest type contributes to the long‐term decline and dampening of fluctuations in *M. rufocanus* (Magnusson, Hörnfeldt, & Ecke, 2015). Relationships between community structure and landscape changes were analyzed using available land cover data from 1970 to 2005, that is, corresponding to the small mammal data in cycles I‐X in 1972‐2006. The western (50 × 100 km) and eastern (50 × 100 km) part of our study area differ in landscape structure and also with respect to the population dynamics of at least the gray‐sided vole (Ecke, Magnussson, & Hörnfeldt, 2013; Hörnfeldt et al., 2006). Therefore, we also distinguished these two regions in the statistical analyses. The diversity components were calculated in the software PAST 3.0 (Hammer, Harper, & Ryan, 2001) except for Sørensen dissimilarity, turnover, and nestedness that were calculated using the package betapart (Baselga & Orme, 2012) in R 3.1.1 (R Development Core Team, 2014). Correlation analyses and polynomial regressions were carried out in Statistica version 12 (StatSoft, 2015) and SIMPER in PAST 3.0 (Hammer et al., 2001).

## 3 RESULTS

In total, we trapped nine small mammal species. These species included the common shrew (*Sorex araneus* Linnaeus, *n* = 1,235), Laxmann's shrew (*Sorex caecutiens* Laxmann, *n* = 115), pygmy shrew (*Sorex minutus* Linnaeus, *n* = 204), *Neomys fodiens* Pennant (*n* = 39), *Myopus schisticolor* (*n* = 395), *M. glareolus* (*n* = 16,923), *M. rufocanus* (*n* = 2,331), *M. agrestis*, (*n* = 2,607), and *Apodemus flavicollis* (*n* = 21).

Alpha, beta, and gamma richness and diversity of all small mammal species combined and of shrews alone showed no consistent temporal trends (Figure 3). In contrast, for voles, richness and diversity at the alpha level, richness at the beta level, and diversity at the gamma level showed significant temporal variation (Figure 3). Vole richness at the alpha level decreased until cycle IX (2000–2002) and has increased since then (Figure 3b), whereas vole richness at the beta level showed the opposite pattern (Figure 3e). Considering only voles and the western and eastern study area separately, alpha richness in the western and eastern part of the study area decreased until cycle IX (2000–2002) and cycle VI (1989–1992), respectively (Figure S1). Beta richness did not differ between the western and eastern part and showed a humped shape temporal trend in the eastern part, while gamma richness decreased permanently with one species in the eastern part in cycle VI (1989–1992) (Figure S1). In contrast, the species pool of voles in the entire study area did not change over time (Figure 3h).

**Figure 4:**
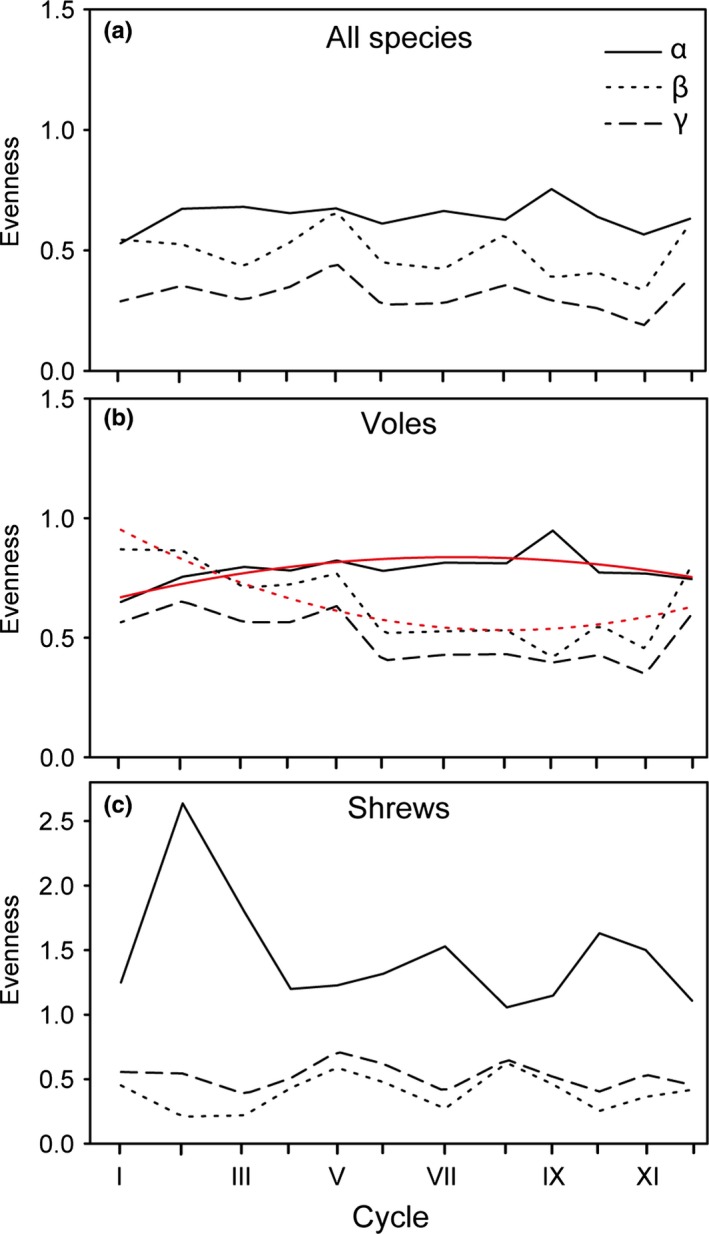
Temporal changes of alpha, beta, and gamma evenness of all species combined (a) and separated for voles (b) and shrews (c) in the 12 cycles (roman numerals) 1972–2012; cycle I: 1972–1975, II: 1976–1979, III: 1980–1982, IV: 1983–1985, V: 1986–1988, VI: 1989–1992, VII: 1993–1996, VIII: 1997–1999, IX: 2000–2002, X: 2003–2005, XI: 2006–2008, and XII: 2009–2012 (cf. Figure 2). Fitted lines indicate significant temporal changes, tested by polynomial regression (see Table S3 for the results from the statistical analyses). Distance between tickmarks on the *x*‐axis reflects relative length of cycle (3 or 4 years; cf. Figure 2)

Richness was always higher than diversity except for shrews at the alpha level where diversity was higher than richness (Figure 3; *T*_12_=0.00, *p *<* *.01 for all comparisons). The higher richness compared to diversity resulted also in low evenness (Figure 4). Alpha and beta evenness of voles, but not of shrews and all species combined, showed long‐term trends, with alpha evenness increasing until cycle IX (2000–2002) and decreasing since then, whereas the opposite was observed for beta evenness (Figure 4).

**Figure 5:**
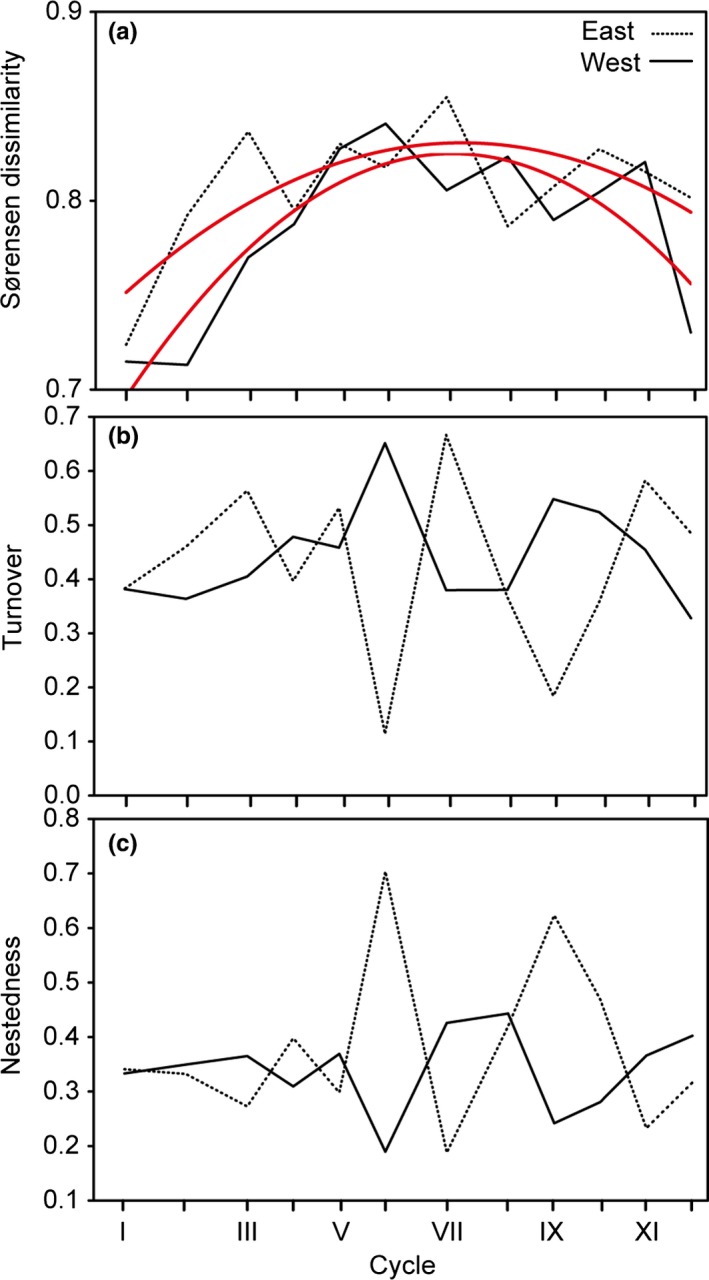
Temporal changes of Sørensen dissimilarity (a), turnover (species replacement; b), and nestedness (species addition/removal; c) of voles in the 12 cycles (roman numerals) 1972–2012; cycle I: 1972–1975, II: 1976–1979, III: 1980–1982, IV: 1983–1985, V: 1986–1988, VI: 1989–1992, VII: 1993–1996, VIII: 1997–1999, IX: 2000–2002, X: 2003–2005, XI: 2006–2008, and XII: 2009–2012 (cf. Figure 2). Fitted lines indicate significant temporal changes, tested by polynomial regression (see Table S4 for the results from the statistical analyses). Distance between tickmarks on the *x*‐axis reflects relative length of cycle (3 or 4 years; cf. Figure 2)

Beta diversity as calculated by Sørensen dissimilarity showed a hump‐shaped trend over time in the eastern and western part of the study area (Figure 5). In contrast, the beta diversity components turnover and nestedness did not show any long‐term trends, but rather fluctuated with high amplitude; turnover generally being higher than nestedness in the western (*T*_12_=14, *p *<* *.05) but not eastern part of the study area (*T*_12_=14, *p *>* *.05) (Figure 5).

**Figure 6:**
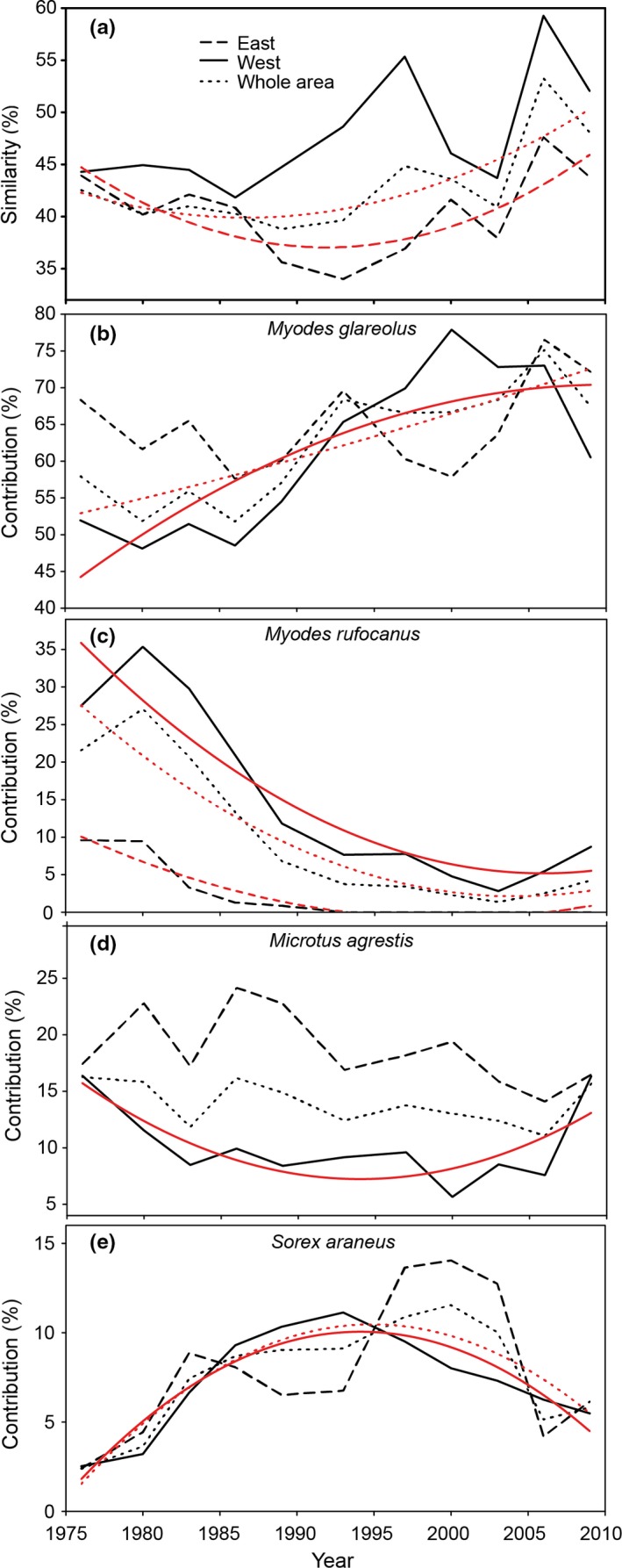
Temporal changes (*n *=* *11 and not 12 due to analysis of temporal changes between consecutive cycles) in (a) similarity between consecutive cycles based on trapping indices and (b–e) contribution (%) of the respective small mammal species to the observed changes in similarity in 12 cycles 1972–2012 (cf. Figure 2). Only small mammals that contributed with at least 10% to the similarity in two consecutive cycles are shown. Fitted lines indicate significant temporal changes, tested by polynomial regression (see Table S5 for the results from the statistical analyses)

In addition to a temporal increase in vole alpha evenness for the whole study area in the 1970s until cycle VIII (1997–2000) (Figure 4b), the cycle‐to‐cycle similarity (100—Sørensen dissimilarity) of pooled density of all small mammal species increased over time in the whole and in the eastern study area, but not in the western study area (Figure 6a). The increase in similarity was most likely due to the increase in relative contribution of *M. glareolus* (especially in the western part of the study area), and to the decrease of *M. agrestis* (in the western part of the study area) and especially of *M. rufocanus* (in the western and eastern part and in the whole study area) (Figure 6b–d). The relative contribution of the shrew *S. araneus* was generally lower than that of the voles *M. glareolus* and *M. agrestis*, but has been higher than that of *M. rufcanus* since the 1990s, especially in the eastern part of the study area (Figure 6).

The community contribution of the bank vole in the western part of the study area was related to the vole's mean density per cycle in autumn (*r*_*s*_ = .71, *n *=* *11, *p *<* *.05). In contrast, in the eastern part of the study area (irrespective of season) and in the western part in spring, the community contribution of the bank vole was not related to the vole's mean density per cycle (*r*_*s*_ < |.54|, *n *=* *11, *p *>* *.05).

**Figure 7:**
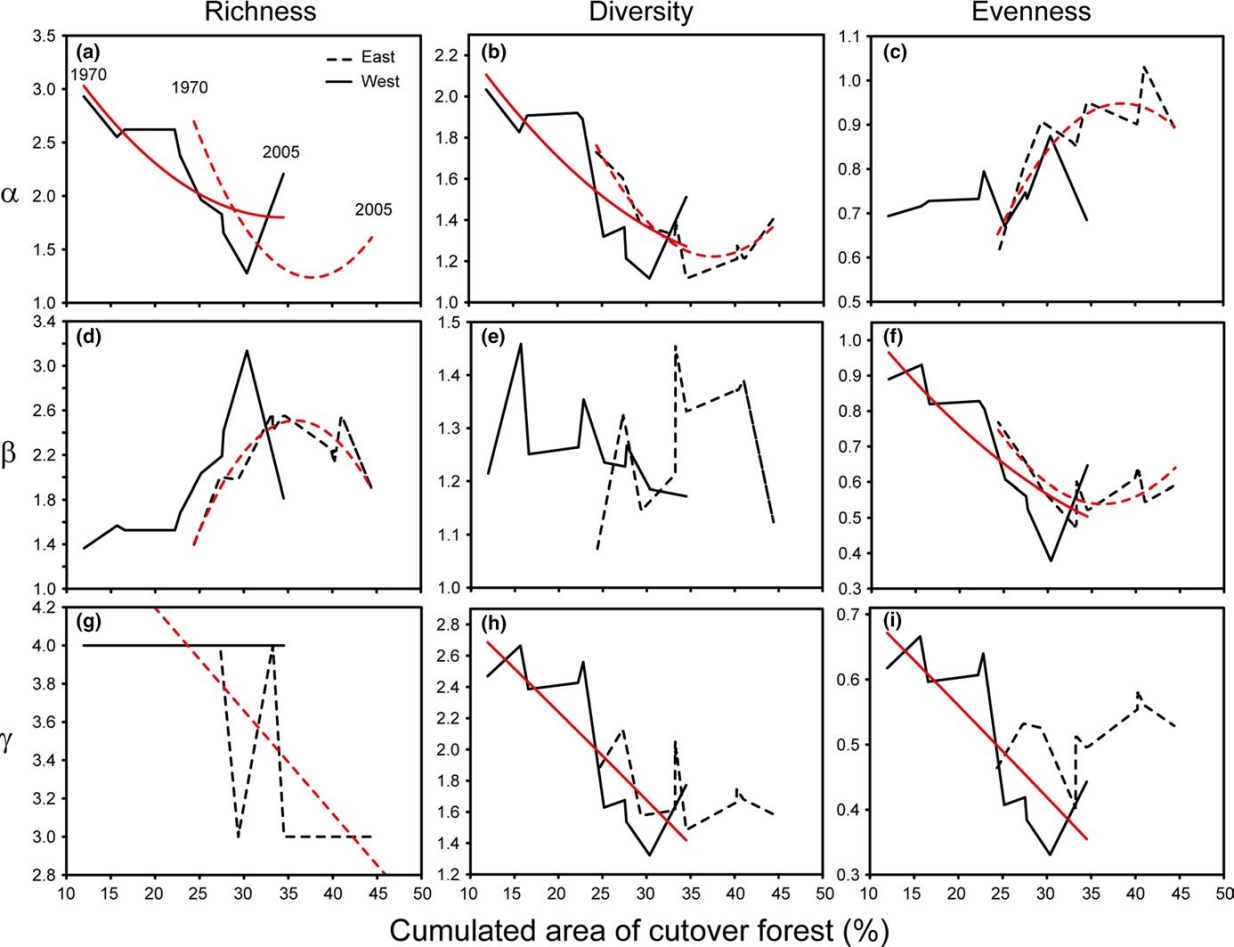
Relationship of alpha (a‐c), beta (d‐f), and gamma (g‐i) richness (a, d, g), diversity (b, e, h) and evenness (c, f, i) of voles in the eastern and western part of the study area with the cumulated proportional area (%) of cutover forest in the landscape for the 10 cycles in 1972–2006, for which landscape data were available. The start and end of the curves indicate the cumulated area of cutover forest in 1970 and 2005, respectively. Fitted lines indicate significant temporal changes, tested by polynomial regression (a–d, f) and linear regression analysis (g‐i) (see Table S6 for the results from the statistical analyses)

Alpha richness and diversity of voles in the western and eastern part of the study area decreased with increased cumulated area of cutover forests at the landscape scale (Figure 7a and b), whereas evenness increased (Figure 7c). Beta richness of voles in the eastern part of the study area showed a humped‐shaped relationship with the area of cutover forests (Figure 7d), and beta evenness decreased with increased area of cutover forest in both parts of the study area (Figure 7f). Gamma diversity and evenness of voles decreased with increased area of cutover forest in the western part of the study area (Figure 7h and i). Negative relationships were normally characterized by an increase in the response variable 2000–2005 (last two data points in the respective lines) despite increased area of cutover forests (Figure 7).

Turnover and nestedness (Figure 5b and c) were neither related to the cumulated area of cutover forest in the western (adj. *r* ^2^ = .05, *F*_2,7_ = 1.23, *p *>* *.05 and adj. *r* ^2^ = −.21, *F*_2,7_=0.22, *p *>* *.05, respectively) nor eastern part of the study area (adj. *r* ^*2*^ = −.24, *F*_2,7_ = .11, *p *>* *.05 and adj. *r* ^2^ = −.15, *F*_2,7_ = .40, *p *>* *.05, respectively).

**Figure 8:**
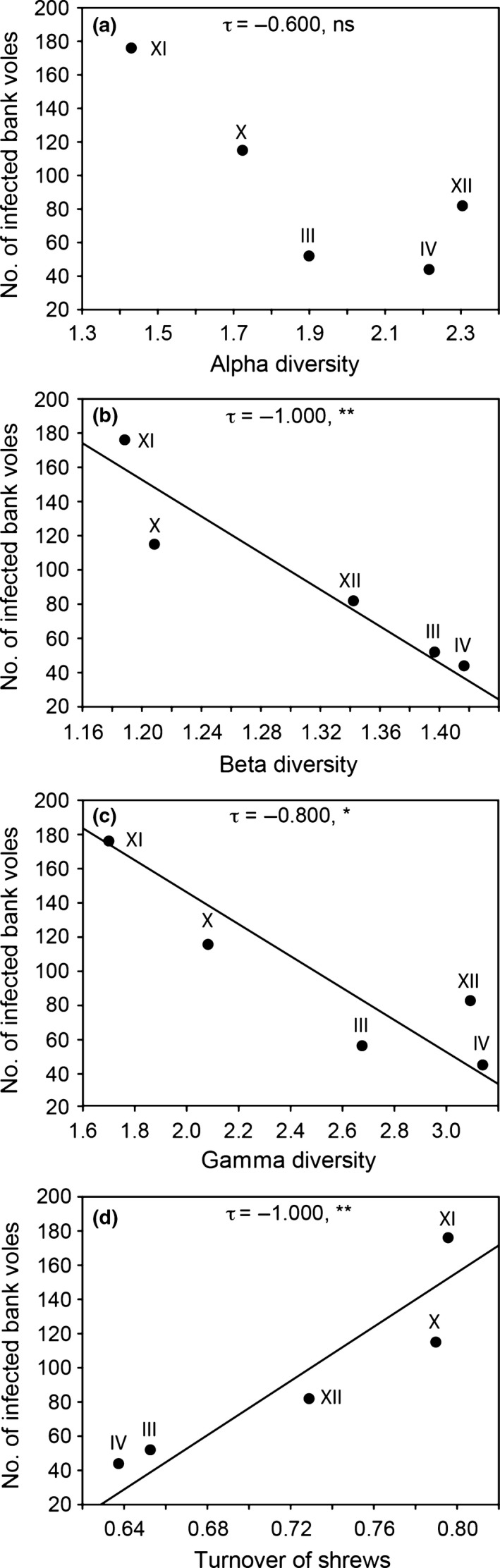
Relationship of the cumulated number of PUUV‐infected bank voles in spring per cycle (III–IV, X–XII) with small mammal alpha (a), beta (b), and gamma (c) diversity, as well as with the beta diversity component turnover of shrews (replaced by vole species) (d). Fitted lines from linear regression analysis indicate significant correlations. Kendall's tau values and significance are given (ns nonsignificant, *p <* *.05 and **p <* *.01)

**Figure 9:**
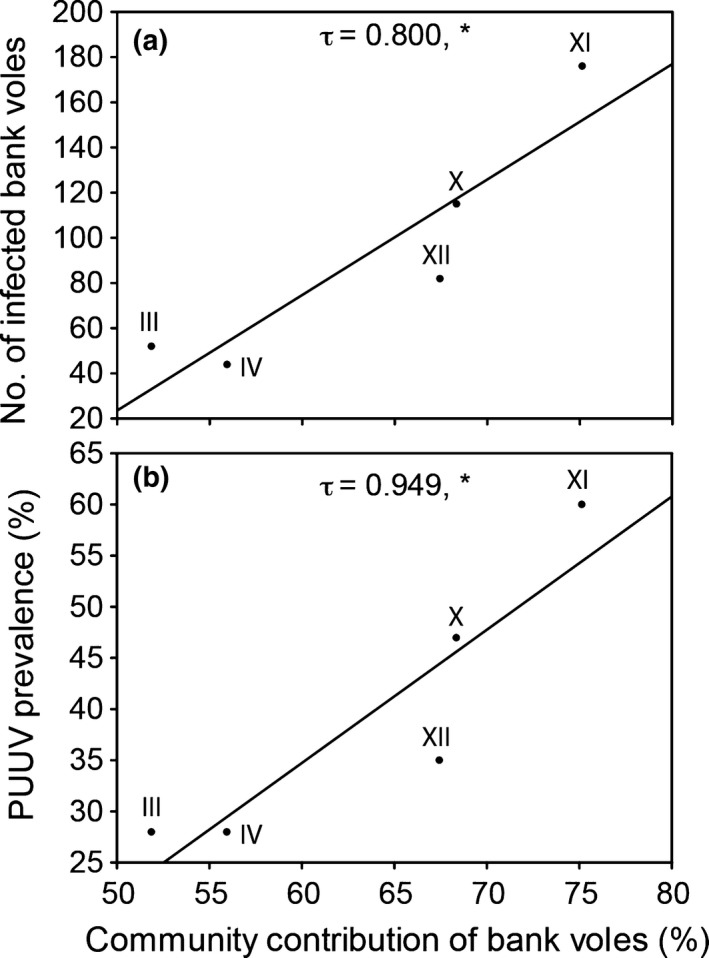
Relationship of the cumulated number of PUUV‐infected bank voles (a) and average PUUV prevalence (b), in spring per cycle (III–IV, X–XII), with the community contribution of the bank vole (*M. glareolus*). Fitted lines from linear regression analysis indicate significant correlations. Kendall's tau values and significance are given (*p <* *.05).

The cumulated number of PUUV‐infected bank voles in spring per cycle was not correlated with alpha diversity, negatively correlated with beta and gamma diversity, and positively correlated with the turnover of shrews (Figure 8). Average PUUV prevalence in spring per cycle was negatively correlated with beta diversity (τ = −0.949, *p *<* *.05), positively correlated with the turnover of shrews (τ = 0.949, *p *<* *.05), but not correlated with alpha and gamma diversity (τ = −0.525, *p *>* *.05 and τ = −0.738, *p *>* *.05, respectively). In addition, in spring, the cumulated number of PUUV‐infected bank voles and average PUUV‐prevalence per cycle was positively correlated with the community contribution of the bank vole (τ = 0.800, *p *<* *.05 and τ = 0.949, *p *<* *.05, respectively) (Figure 9).

## 4 DISCUSSION

Dampened or even collapsing vole and lemming cycles are expected to have profound effects for ecosystem functioning including trophic interactions (Hörnfeldt et al., 2005; Ims et al., 2008; Olofsson et al., 2012; Schmidt et al., 2012). In addition to previously reported long‐term changes in the population dynamics and densities of microtine rodents in Europe (Cornulier et al., 2013; Gouveia et al., 2015; Hörnfeldt, 2004; Ims et al., 2008), our analyses, for the first time, as far as we know, addressed the profound changes in community structure of small mammals by analyzing a range of quantitative metrics. The here observed changes in northern Sweden in 1971–2013 were apparent in diversity, richness, and evenness of especially the three vole species (*M. glareolus*,* M. rufocanus* and *M. agrestis*) at the alpha, beta, and gamma level. In addition, also *S. araneus* contributed significantly to the community structure, especially in the eastern part of the study area. Despite the observed profound changes in population dynamics of the studied species, including local disappearance of *M. rufocanus* (Magnusson, Hörnfeldt, & Ecke, 2015), no species has disappeared at the regional level as revealed by gamma species richness.

Small mammal community changes are not a new phenomenon. Blois, Mcguire, and Hadly (2010) have reported local diversity loss including decline in evenness and turnover in a small mammal community in northern California, USA, as a response to late‐Pleistocene climate change. Interestingly, the decline in evenness was driven by an increase in the abundance of deer mice (*Peromyscus* spp.) (Blois et al., 2010), a habitat generalist like *M. glareolus* (Gurnell, 1985; Wywialowski, 1987).

Among voles, the bank vole was the overall dominating species, even though also this species significantly declined in density until the early 1990s, resulting in low community evenness and consequently higher richness than diversity. Low evenness or the dominance by one or a few species results in reduced resistance in the stability of community functioning to environmental stress (Wittebolle et al., 2009). In our study, evenness among voles at the alpha level increased until cycle IX (2000–2002), a trend that was expressed by the decrease in the community contribution of *M. rufocanus* and the increase in *M. glareolus*.

Most likely, the long‐term depression of mean densities of the habitat specialist *M. rufocanus* is caused by forestry‐induced habitat loss (Magnusson, Hörnfeldt, & Ecke, 2015). The cause of the long‐term depression of mean densities of the field vole, followed by at least temporal recovery in the last cycle (XII; 2009–2012), is unknown, but climatic factors might be involved (Magnusson, Ecke, et al., 2015). The increased incidence of cutover forests was probably also an important contributing factor to the observed changes of richness, diversity, and evenness at the alpha, beta, and gamma level in our study. Forest management in northern Sweden is operating at the scale of forest patches with profound ecological effects at different spatial scales ranging from patch to region (Ecke, Magnussson, & Hörnfeldt, 2013). As turnover in our vole community was generally higher than nestedness in the western part of the study area, according to theory (Wright & Reeves, 1992), there would be a need for a regional landscape management approach to reverse the decline in vole richness and diversity in 1972–2005. Interestingly, alpha richness and diversity of voles showed an increasing trend during the last two cycles, that is, in 2006–2012. In this study, we had only access to land cover data until 2005. In future studies, it is important to investigate and consider changes in habitat quality of cutover forests and temporal changes in forest management practices. Gradually, the oldest cutover forests (mainly cut in the 1970s but some already in the 1950s) reach habitat quality similar to that found in old forests and that is suitable to sustain high small mammal richness and diversity. Indeed, species richness and abundance of small mammals change during forest succession (Ecke, Löfgren, & Sörlin, 2002).

In a recent study, Korpela et al. (2013) studied the effect of climate on population dynamics of voles. They calculated vole density by pooling the trapping index of all vole species, including *M. glareolus*,* M. rufocanus,* and *M. agrestis,* although they occupy different ecological niches. Our results suggest that it is important to consider both the community but also the species level when analyzing responses of small mammals to environmental change.

### 4.1 Implications for infectious diseases

The observed long‐term dynamics of biodiversity change of small mammal communities have implications for infectious diseases and their local and regional management. The bank vole is a habitat generalist but largely a forest dwelling species (Johannesen & Mauritzen, 1999) that occasionally reaches high densities even in open habitats such as clear‐cuts (Ecke, Löfgren, & Sörlin, 2002). Both *M. rufocanus* and *M. agrestis* are competitively superior to the more common bank vole (Hanski & Henttonen, 1996; Henttonen, Kaikusalo, Tast, & Viitala, 1977; Johannesen & Mauritzen, 1999). The release of competition from *M. rufocanus* and *M. agrestis* and potentially also from relaxed predation by the decline of Tengmalm's owl (*Aegolius funereus*) (Hörnfeldt et al., 2005; Khalil et al., 2016) may explain the increased community contribution by *M. glareolus*. The increased contribution of *M. glareolus* that was largely independent of its density and that occurred despite a long‐term decline of this species until the 1990s might also explain the increase in PUUV prevalence. This is not surprising as pathogen prevalence can be independent of host density via diversity‐driven reduction in encounter rate (e.g., Clay, Lehmer, St Jeor, et al., 2009b; Keesing, Holt, & Ostfeld, 2006), even though pathogen prevalence and number of infected hosts are generally related to overall host abundance (reviewed by Khalil, Hornfeldt, et al., 2014). Our results suggest that a more simplified small mammal community dominated by *M. glareolus* increases risk for humans (see also Khalil et al., 2016). Vole cycle XI (starting in spring 2006 and ending in spring 2009) was overall characterized by the highest density (except for cycle I; Figure 2) and high community contribution of *M. glareolus* (Figure 6), the highest number of PUUV‐infected *M. glareolus* and the highest PUUV prevalence (Figure 9). Interestingly, this is also the cycle when the so far highest number of human cases of NE was reported in Sweden (Olsson, Hjertqvist, Lundkvist, & Hörnfeldt, 2009). Future studies should investigate whether the here identified effects of a deteriorated small mammal community decrease the resistance of the forest landscape to outbreaks of zoonotic diseases in general and of PUUV and the associated NE in particular.

According to the dilution effect, a decrease in diversity leads to an increase in disease risk through either increasing host abundance (susceptible host regulation) or increase in host contact rate (encounter increase) (sensu Keesing et al., 2006). Indeed, we identified overall negative relationships between small mammal diversity and disease risk. Most recently, Khalil et al. (2016) found that infection probability in *M. glareolus* decreases when densities of *S. araneus* increase. In our study, both the number of PUUV‐infected *M. glareolus* and PUUV prevalence appeared to increase when the replacement (turnover) of shrew species (replaced by voles) among sites increased. This latter result supports the most recently suggested regulatory role of shrews for disease risk by “encounter reduction” (see Keesing et al., 2006 for reduction concepts), that is, potential shrew‐induced reduced contact rates among *M. glareolus* (Khalil et al., 2016). Our results indicate the presence of a dilution effect in the study system and pose a rare opportunity to test in detail the concept and related mechanisms of the dilution effect on PUUV to be analyzed in biobanked *M. glareolus* specimens (Odsjö, 2006; Hörnfeldt, 2015) for the entire period 1979 to present, that is, the time period covering the here presented study. A major societal challenge is, however, to apply results on the importance of biodiversity for health to public policy (Keesing & Ostfeld, 2015).

The correlation between beta diversity (including turnover of shrews) and cumulated number of PUUV infected bank voles in spring per cycle indicates that processes beyond those occurring at the local scale are influencing disease dynamics. For PUUV to be spread into new areas or to recolonize former areas, host movement is important; a movement that most likely is affected by changes in landscape structure, that is, processes occurring at the spatial scale relevant for beta diversity. Our results strengthen the importance of a multilevel approach (ranging from individual species to the whole community) when studying and trying to understand the ecological implications of temporal changes in the population dynamics of small mammals and associated disease dynamics.

## CONFLICT OF INTEREST

None declared.
