# Supplementary material for: Dampening of population cycles in voles affects small mammal community structure, decreases diversity, and increases prevalence of a zoonotic disease
Source: Ecol Evol. 2017 Jun 9;7(14):5331–42. doi: 10.1002/ece3.3074 (PMC5528244; doi:10.1002/ece3.3074)
Supplement: Supplementary file 1 [file ECE3-7-5331-s001.docx]

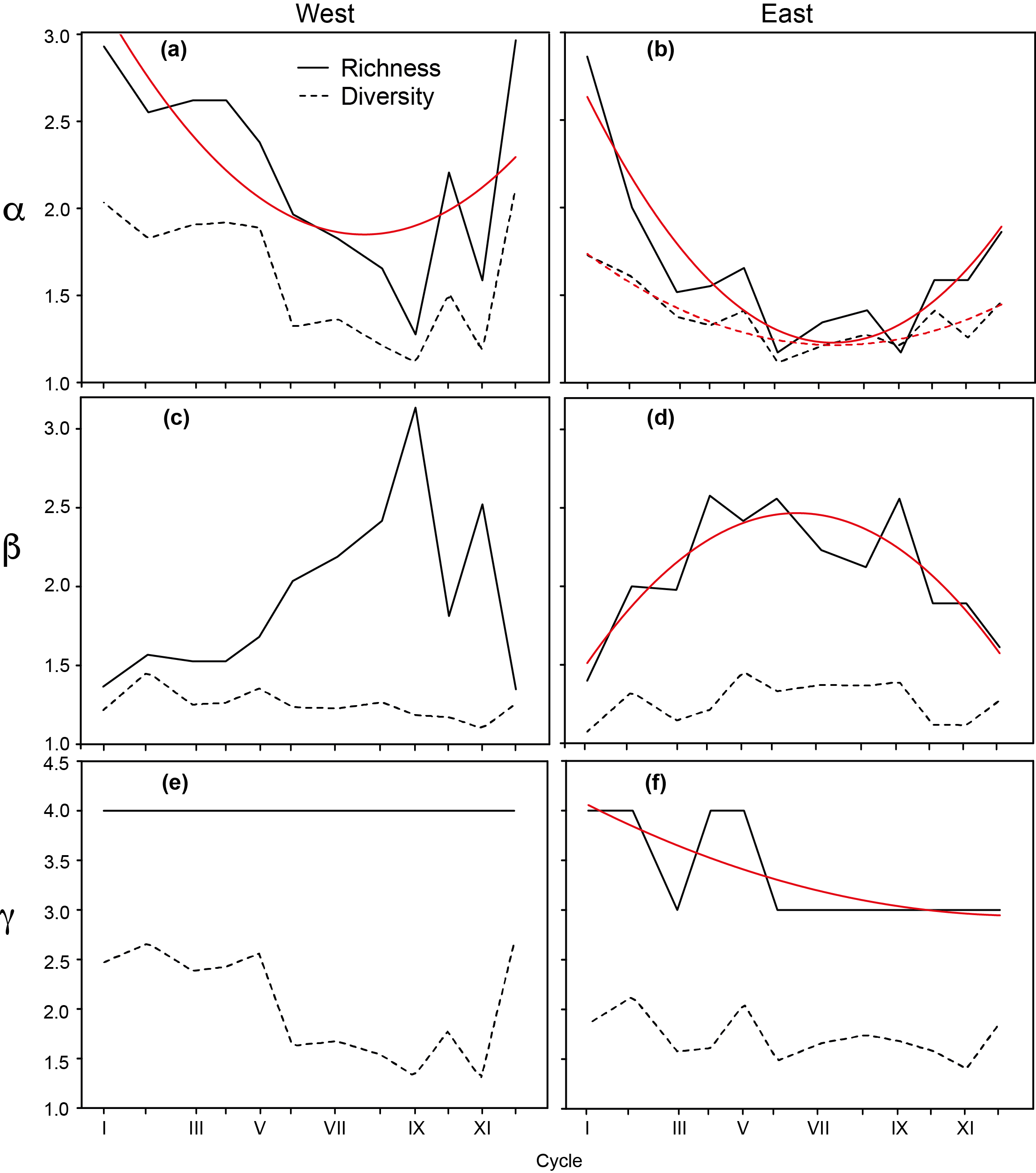


**Figure S1**. Temporal changes of alpha, beta and gamma richness and diversity of voles in the 12 cycles (roman numerals) 1972-2012 in the western and eastern part of the study area; cycle I: 1972-1975, II: 1976-1979, III: 1980-1982, IV: 1983-1985, V: 1986-1988, VI: 1989-1992, VII: 1993-1996, VIII: 1997-1999, IX: 2000-2002, X: 2003-2005, XI: 2006-2008 and XII: 2009-2012 (cf. Fig. 1). Fitted lines indicate significant temporal changes, tested by polynomial regression. Alpha richness, west: adjusted *R*^2^ = 0.41, *F*_2, 9_ = 4.84, *p*<0.05; alpha richness, east: adjusted *R*^2^ = 0.83, *F*_2, 9_ = 27.77, *p*<0.001; alpha diversity, east: adjusted *R*^2^ = 0.76, *F*_2, 9_ = 18.25, *p*<0.001; beta richness, east: adjusted *R*^2^ = 0.70, *F*_2, 9_ = 14.10, *p*<0.001; gamma richness, east: adjusted *R*^2^ = 0.46, *F*_2, 9_ = 5.73, *p*<0.05. All other relationships were non-significant (*F*_2, 9_ < 4.90, *p*>0.05). Distance between tickmarks on the x-axis reflects relative length of cycle (3 or 4 years; cf. Fig. 1). See also Table below.

**Table for Fig. S1.** Results from polynomial regression analysis (Adjusted *R*^2^) between richness and diversity, respectively, and vole cycle number (*n* = 12), at the alpha, beta and gamma level and divided into eastern and western study area. See also Fig. S1.

| Response variable | Adjusted *R*^2^ | *p* |
| --- | --- | --- |
| Alpha level |  |  |
| Richness - East | 0.83 | 0.000 |
| Richness - West | 0.41 | 0.037 |
| Diversity - East | 0.76 | 0.000 |
| Diversity - West | 0.35 | 0.058 |
| Beta level |  |  |
| Richness - East | 0.70 | 0.002 |
| Richness - West | 0.28 | 0.091 |
| Diversity - East | 0.24 | 0.119 |
| Diversity - West | 0.16 | 0.190 |
| Gamma level |  |  |
| Richness - East | 0.46 | 0.025 |
| Richness – West | - | - |
| Diversity – East | 0.04 | 0.339 |
| Diversity - West | 0.33 | 0.064 |
