# Supplementary material for: Dampening of population cycles in voles affects small mammal community structure, decreases diversity, and increases prevalence of a zoonotic disease
Source: Ecol Evol. 2017 Jun 9;7(14):5331–42. doi: 10.1002/ece3.3074 (PMC5528244; doi:10.1002/ece3.3074)
Supplement: Supplementary file 2 [file ECE3-7-5331-s002.docx]

**Table S1.** Results from time series trend analysis to identify significant changes in the trapping index of the three vole species (*M. glareolus*, *M. rufocanus* and *M. agrestis*) studied in 1971-2012. As a first step, we divided the time series into spring and autumn data. Then, a Fourier analysis identified the cyclicity of all the time series to be three years (>2 years frequency). In the time series analyses, we first decomposed the series into its seasonal pattern (to account for its cyclic nature), general trend, and noise. Then we used the trend component to analyse long-term changes in the time-series using a linear model with first and second order polynomials. In all cases, the AIC (Akaike’s Information Criterion) was lower when a second order polynomial describing the changes with time was added. See also Fig. 2.

| Species and season | Parameter | Estimate | Std. error | *t*-value | *P*-value |
| --- | --- | --- | --- | --- | --- |
| *M. glareolus* (spring) | Intercept | 1.377 × 10^4^ | 2.182 × 10^3^ | 6.313 | *** |
|  | Year | ‐1.382 × 10^1^ | 2.190 × 10^0^ | ‐6.309 | *** |
|  | Year^2^ | 3.466 × 10^-3^ | 5.498 × 10^-4^ | 6.304 | *** |
| *M. glareolus* (autumn) | Intercept | 3.271 × 10^4^ | 6.910 × 10^3^ | 4.733 | *** |
|  | Year | ‐3.283 × 10^1^ | 6.938 × 10^0^ | ‐4.732 | *** |
|  | Year^2^ | 8.239 × 10^-3^ | 1.741 × 10^-3^ | 4.731 | *** |
|  |  |  |  |  |  |
| *M. rufocanus* (spring) | Intercept | 4.239 × 10^3^ | 6.293 × 10^2^ | 6.736 | *** |
|  | Year | ‐4.238 × 10^0^ | 6.318 × 10^-1^ | ‐6.708 | *** |
|  | Year^2^ | 1.059 × 10^-3^ | 1.586 × 10^-4^ | 6.681 | *** |
| *M. rufocanus* (autumn) | Intercept | 9.034 × 10^3^ | 1.044 × 10^3^ | 8.652 | *** |
|  | Year | ‐9.033 × 10^0^ | 1.048 × 10^0^ | ‐8.616 | *** |
|  | Year^2^ | 2.258 × 10^-3^ | 2.632× 10^-4^ | 8.580 | *** |
|  |  |  |  |  |  |
| *M. agrestis* (spring) | Intercept | 7.170 × 10^3^ | 9.506 × 10^2^ | 7.543 | *** |
|  | Year | ‐7.185 × 10^0^ | 9.544 × 10^-1^ | ‐7.528 | *** |
|  | Year^2^ | 1.800 × 10^-3^ | 2.396 × 10^-4^ | 7.514 | *** |
| *M. agrestis* (autumn) | Intercept | 4.619 × 10^3^ | 9.954 × 10^2^ | 4.641 | *** |
|  | Year | ‐4.636 × 10^0^ | 9.994 × 10^-1^ | ‐4.639 | *** |
|  | Year^2^ | 1.163 × 10^-3^ | 2.509 × 10^-4^ | 4.637 | *** |

The analyses were performed in R (R Core Team 2016) using TSA (Kung-Sik and Ripley 2012) and base packages.

Kung-Sik Chan and Brian Ripley (2012). TSA: Time Series Analysis. R package version 1.01. URL https://CRAN.R-project.org/package=TSA.

R Core Team (2016). R: A language and environment for statistical computing. R Foundation for Statistical Computing, Vienna, Austria. URL https://www.R-project.org/.

**Table S2.** Results from polynomial regression analysis (Adjusted *R*^2^) and correlation analyses (*r_s_*) between richness and diversity, respectively, and vole cycle number (*n* = 12) at the alpha, beta and gamma level. ** *p*<0.01. See also Fig. 3.

| Response variable | Adjusted *R*^2^ | *r_s_* | *p* |
| --- | --- | --- | --- |
| Alpha level |  |  |  |
| All species - Richness | 0.22 |  | 0.134 |
| All species - Diversity | -0.06 |  | 0.533 |
| Voles - Richness | 0.73 |  | 0.001 |
| Voles - Diversity | 0.54 |  | 0.012 |
| Shrews - Richness | -0.05 |  | 0.512 |
| Shrews - Diversity | 0.05 |  | 0.323 |
| Beta level |  |  |  |
| All species - Richness | -0.04 |  | 0.494 |
| All species - Diversity | 0.23 |  | 0.124 |
| Voles - Richness | 0.52 |  | 0.015 |
| Voles - Diversity | 0.16 |  | 0.181 |
| Shrews – Richness | -0.04 |  | 0.484 |
| Shrews - Diversity | 0.06 |  | 0.300 |
| Gamma level |  |  |  |
| All species - Richness | -0.11 |  | 0.657 |
| All species - Diversity | -0.04 |  | 0.493 |
| Voles - Richness | 0.30 |  | 0.079 |
| Voles - Diversity |  | -0.43 | ** |
| Shrews - Richness | -0.10 |  | 0.629 |
| Shrews - Diversity | -0.21 |  | 0.967 |

**Table S3.** Results from polynomial regression analyses between evenness and vole cycle number (*n* = 12) at the alpha, beta and gamma level, respectively, for all species combined as well as separately for voles and shrews. See also Fig. 4.

| Response variable | Adjusted *R*^2^ | *p* |
| --- | --- | --- |
| All species combined |  |  |
| Alpha | 0.24 | 0.118 |
| Beta | -0.10 | 0.613 |
| Gamma | 0.06 | 0.311 |
| Voles |  |  |
| Alpha | 0.67 | 0.003 |
| Beta | 0.48 | 0.022 |
| Gamma | 0.07 | 0.291 |
| Shrews |  |  |
| Alpha | 0.04 | 0.335 |
| Beta | -0.11 | 0.645 |
| Gamma | -0.06 | 0.532 |

**Table S4.** Results from polynomial regression analyses between Sørensen dissimilarity, turnover and nestedness of voles, respectively, and vole cycle number (*n* = 12) in the eastern and western part of the study area. See also Fig. 5).

| Response variable | Adjusted *R*^2^ | *p* |
| --- | --- | --- |
| Sørensen dissimilarity |  |  |
| East | 0.41 | 0.037 |
| West | 0.71 | 0.002 |
| Turnover |  |  |
| East | -0.19 | 0.891 |
| West | 0.10 | 0.257 |
| Nestedness |  |  |
| East | -0.10 | 0.627 |
| West | -0.19 | 0.871 |

**Table S5.** Results from polynomial regression analyses between Similarity, and the contribution of the respective species, respectively, and vole cycle number (*n* = 12) in the whole study area as well as in the eastern and western part of the study area. See also Fig. 6.

| Response variable | Adjusted *R*^2^ | *p* |
| --- | --- | --- |
| Similarity |  |  |
| Whole study area | 0.55 | 0.017 |
| East | 0.47 | 0.033 |
| West | 0.26 | 0.123 |
| Contribution of *M. glareolus* |  |  |
| Whole study area | 0.64 | 0.007 |
| East | 0.33 | 0.084 |
| West | 0.57 | 0.013 |
| Contribution of *M. rufocanus* |  |  |
| Whole study area | 0.84 | 0.000 |
| East | 0.87 | 0.000 |
| West | 0.80 | 0.001 |
| Contribution of *M. agrestis* |  |  |
| Whole study area | 0.14 | 0.225 |
| East | 0.28 | 0.112 |
| West | 0.53 | 0.020 |
| Contribution of *S. araneus* |  |  |
| Whole study area | 0.80 | 0.001 |
| East | 0.37 | 0.064 |
| West | 0.84 | 0.000 |

**Table S6.** Results from polynomial regression analysis (Adjusted *R*^2^) and correlation analyses (*r_s_*) between richness, diversity and evenness, respectively, and the cumulated area of cut-over forests in the study area (*n* = 10) at the alpha, beta and gamma level and divided into eastern and western study area. * *p*<0.05. See Fig. 7.

| Response variable | Adjusted *R*^2^ | *r_s_* | *p* |
| --- | --- | --- | --- |
| Alpha level |  |  |  |
| Richness - East | 0.84 |  | 0.001 |
| Richness - West | 0.54 |  | 0.027 |
| Diversity - East | 0.79 |  | 0.002 |
| Diversity - West | 0.51 |  | 0.034 |
| Evenness – East | 0.66 |  | 0.009 |
| Evenness - West | -0.12 |  | 0.611 |
| Beta level |  |  |  |
| Richness - East | 0.80 |  | 0.002 |
| Richness - West | 0.32 |  | 0.106 |
| Diversity - East | 0.34 |  | 0.098 |
| Diversity - West | 0.19 |  | 0.195 |
| Evenness – East | 0.50 |  | 0.035 |
| Evenness - West | 0.56 |  | 0.023 |
| Gamma level |  |  |  |
| Richness - East |  | -0.72 | * |
| Richness – West | 0.32 |  | 0.108 |
| Diversity – East | -0.01 |  | 0.422 |
| Diversity - West |  | -0.76 | * |
| Evenness – East | 0.05 |  | 0.353 |
| Evenness - West |  | -0.76 | * |
